# Dichlorido(2,3-di-2-pyridyl­pyrazine-κ^2^ *N* ^2^,*N* ^3^)palladium(II)

**DOI:** 10.1107/S1600536811043753

**Published:** 2011-10-29

**Authors:** Kwang Ha

**Affiliations:** a School of Applied Chemical Engineering, The Research Institute of Catalysis, Chonnam National University, Gwangju 500-757, Republic of Korea

## Abstract

The Pd^II^ ion in the title complex, [PdCl_2_(C_14_H_10_N_4_)], has a slightly distorted square-planar environment defined by the two pyridine N atoms of the chelating 2,3-di-2-pyridyl­pyrazine ligand and two chloride anions. The pyridine rings are considerably inclined to the least-squares plane of the PdCl_2_N_2_ unit [maximum deviation = 0.073 (1) Å], with dihedral angles of 64.19 (9) and 66.55 (9)°. The pyrazine ring is almost perpendicular to this plane and the dihedral angle is 88.2 (1)°. Two independent inter­molecular C—H⋯Cl hydrogen bonds, both involving the same Cl atom as a hydrogen-bond acceptor, give rise to chains running along the *a* and *b* axes, generating a layer structure extending parallel to (001). Mol­ecules are stacked in columns along the *a* axis. Along the *b* axis, successive mol­ecules stack in opposite directions.

## Related literature

For the structure of the isotypic [PtBr_2_(2,3-di-2-pyridyl­pyrazine)] analog, see: Ha (2011 ▶). For related Pt^II^ complexes, see: Granifo *et al.* (2000 ▶); Cai *et al.* (2009 ▶).
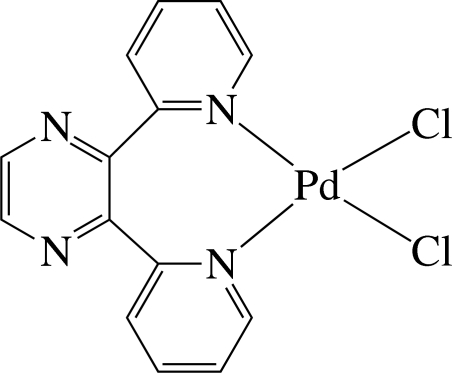


## Experimental

### 

#### Crystal data

[PdCl_2_(C_14_H_10_N_4_)]
*M* *_r_* = 411.56
Monoclinic, 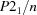

*a* = 8.3414 (9) Å
*b* = 15.3270 (16) Å
*c* = 11.7208 (12) Å
β = 101.027 (2)°
*V* = 1470.8 (3) Å^3^
*Z* = 4
Mo *K*α radiation
μ = 1.62 mm^−1^
*T* = 200 K
0.28 × 0.26 × 0.20 mm

#### Data collection

Bruker SMART 1000 CCD diffractometer
Absorption correction: multi-scan (*SADABS*; Bruker, 2000 ▶) *T*_min_ = 0.864, *T*_max_ = 1.000
10347 measured reflections
3540 independent reflections
2864 reflections with *I* > 2σ(*I*)
*R*_int_ = 0.030

#### Refinement

*R*[*F* ^2^ > 2σ(*F* ^2^)] = 0.028
*wR*(*F* ^2^) = 0.075
*S* = 1.18
3540 reflections
190 parameters
H-atom parameters constrained
Δρ_max_ = 1.00 e Å^−3^
Δρ_min_ = −0.72 e Å^−3^

### 

Data collection: *SMART* (Bruker, 2000 ▶); cell refinement: *SAINT* (Bruker, 2000 ▶); data reduction: *SAINT*; program(s) used to solve structure: *SHELXS97* (Sheldrick, 2008 ▶); program(s) used to refine structure: *SHELXL97* (Sheldrick, 2008 ▶); molecular graphics: *ORTEP-3* (Farrugia, 1997 ▶) and *PLATON* (Spek, 2009 ▶); software used to prepare material for publication: *SHELXL97*.

## Supplementary Material

Crystal structure: contains datablock(s) global, I. DOI: 10.1107/S1600536811043753/ng5256sup1.cif

Structure factors: contains datablock(s) I. DOI: 10.1107/S1600536811043753/ng5256Isup2.hkl

Additional supplementary materials:  crystallographic information; 3D view; checkCIF report

## Figures and Tables

**Table:** 

Pd1—N3	2.022 (3)
Pd1—N4	2.026 (3)
Pd1—Cl1	2.2939 (10)
Pd1—Cl2	2.3037 (9)

**Table:** 

N3—Pd1—N4	87.89 (12)
Cl1—Pd1—Cl2	93.19 (4)

**Table 2:** Hydrogen-bond geometry (Å, °)

*D*—H⋯*A*	*D*—H	H⋯*A*	*D*⋯*A*	*D*—H⋯*A*
C6—H6⋯Cl1^i^	0.95	2.83	3.445 (4)	124
C11—H11⋯Cl1^ii^	0.95	2.82	3.629 (4)	143

Symmetry codes: (i) 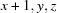; (ii) 
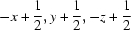
.

**Fig. 1.**
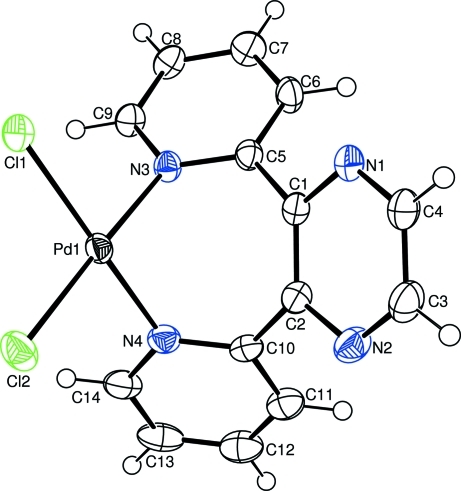
The structure of the title complex, with displacement ellipsoids drawn at the 50% probability level; H atoms are shown as small circles of arbitrary radius.

**Fig. 2.**
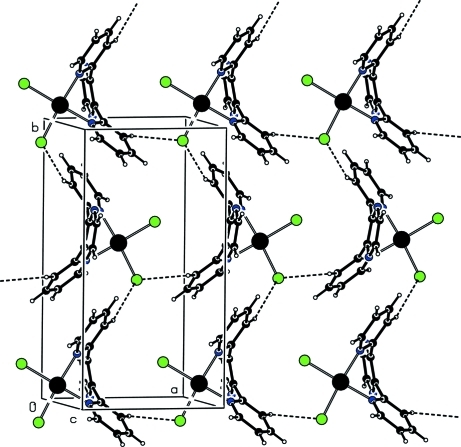
View of the hydrogen-bond interactions of the title complex. Hydrogen-bonds are drawn with dashed lines.

## supplementary crystallographic information

### Comment

The title complex, [PdCl_2_(dpp)] (dpp is 2,3-di-2-pyridylpyrazine, C_14_H_10_N_4_), is isomorphous with the yellow form of [PtBr_2_(dpp)] (Ha, 2011). The Pd^II^ ion has a slightly distorted square-planar environment defined by the two pyridyl N atoms of the chelating dpp ligand and two chloride anions (Fig. 1). The coordination mode of the dpp ligand is similar to that found in the mononuclear Pt(II) complexes [PtCl_2_(dpq)] (dpq = 2,3-di-2-pyridylquinoxaline) (Granifo *et al.*, 2000) and [PtCl_2_(dcdpp)] (dcdpp = 2,3-dicyano-5,6-di-2-pyridylpyrazine) (Cai *et al.*, 2009).

The N3—Pd1—N4 chelate angle of 87.89 (12)° and Cl—Cl repelling contribute the distortion of square, and therefore the *trans* axes are slightly bent [<Cl1—Pd1—N4 = 173.45 (8)° and <Cl2—Pd1—N3 = 178.07 (8)°]. The Pd—N and Pd—Cl bond lengths are nearly equivalent, respectively (Table 1). In the crystal, the two pyridyl rings are considerably inclined to the least-squares plane of the PdCl_2_N_2_ unit [maximum deviation = 0.073 (1) Å] with dihedral angles of 64.19 (9)° and 66.55 (9)°, respectively. The nearly planar pyrazine ring [maximum deviation = 0.021 (3) Å] is almost perpendicular to the unit plane with a dihedral angle of 88.2 (1)°. The dihedral angle between the two pyridyl rings is 80.1 (1)°. Two independent intermolecular C—H···Cl hydrogen bonds, both involving the same Cl atom as an H-bond acceptor, give rise to chains running along the a and b axes, forming a layer structure extending parallel to the *ab* plane (Fig. 2 and Table 2). The complexes are stacked in columns along the *a* axis. When viewed down the *b* axis, the successive complexes stack in the opposite direction. In the columns, numerous inter- and intramolecular π-π interactions between the six-membered rings are present, the shortest ring centroid-centroid distance being 3.732 (2) Å.

### Experimental

To a solution of Na_2_PdCl_4_ (0.2960 g, 1.006 mmol) in MeOH (30 ml) was added 2,3-di-2-pyridylpyrazine (0.2361 g, 1.008 mmol) and stirred for 20 h at room temperature. The formed precipitate was separated by filtration, washed with MeOH, and dried at 50 °C, to give a yellow powder (0.3560 g). Crystals were obtained by slow evaporation from a CH_3_CN solution.

### Refinement

H atoms were positioned geometrically and allowed to ride on their respective parent atoms [C—H = 0.95 Å and *U*_iso_(H) = 1.2*U*_eq_(C)]. The highest peak (1.00 e Å^-3^) and the deepest hole (-0.72 e Å^-3^) in the difference Fourier map are located 1.12 Å and 0.78 Å from the atoms H11 and Pd1, respectively.

### Crystal data

**Table:**

[PdCl_2_(C_14_H_10_N_4_)]	*F*(000) = 808
*M**_r_* = 411.56	*D*_x_ = 1.859 Mg m^−^^3^
Monoclinic, *P*2_1_/*n*	Mo *K*α radiation, λ = 0.71073 Å
Hall symbol: -P 2yn	Cell parameters from 5882 reflections
*a* = 8.3414 (9) Å	θ = 2.2–28.2°
*b* = 15.3270 (16) Å	µ = 1.62 mm^−^^1^
*c* = 11.7208 (12) Å	*T* = 200 K
β = 101.027 (2)°	Block, yellow
*V* = 1470.8 (3) Å^3^	0.28 × 0.26 × 0.20 mm
*Z* = 4	

### Data collection

**Table:**

Bruker SMART 1000 CCD diffractometer	3540 independent reflections
Radiation source: fine-focus sealed tube	2864 reflections with *I* > 2σ(*I*)
graphite	*R*_int_ = 0.030
φ and ω scans	θ_max_ = 28.3°, θ_min_ = 2.2°
Absorption correction: multi-scan (*SADABS*; Bruker, 2000)	*h* = −8→11
*T*_min_ = 0.864, *T*_max_ = 1.000	*k* = −20→18
10347 measured reflections	*l* = −15→15

### Refinement

**Table:**

Refinement on *F*^2^	Primary atom site location: structure-invariant direct methods
Least-squares matrix: full	Secondary atom site location: difference Fourier map
*R*[*F*^2^ > 2σ(*F*^2^)] = 0.028	Hydrogen site location: inferred from neighbouring sites
*wR*(*F*^2^) = 0.075	H-atom parameters constrained
*S* = 1.18	*w* = 1/[σ^2^(*F*_o_^2^) + (0.014*P*)^2^ + 2.5736*P*] where *P* = (*F*_o_^2^ + 2*F*_c_^2^)/3
3540 reflections	(Δ/σ)_max_ < 0.001
190 parameters	Δρ_max_ = 1.00 e Å^−^^3^
0 restraints	Δρ_min_ = −0.72 e Å^−^^3^

### Special details

**Table:**

Geometry. All e.s.d.'s (except the e.s.d. in the dihedral angle between two l.s. planes) are estimated using the full covariance matrix. The cell e.s.d.'s are taken into account individually in the estimation of e.s.d.'s in distances, angles and torsion angles; correlations between e.s.d.'s in cell parameters are only used when they are defined by crystal symmetry. An approximate (isotropic) treatment of cell e.s.d.'s is used for estimating e.s.d.'s involving l.s. planes.
Refinement. Refinement of *F*^2^ against ALL reflections. The weighted *R*-factor *wR* and goodness of fit *S* are based on *F*^2^, conventional *R*-factors *R* are based on *F*, with *F* set to zero for negative *F*^2^. The threshold expression of *F*^2^ > σ(*F*^2^) is used only for calculating *R*-factors(gt) *etc*. and is not relevant to the choice of reflections for refinement. *R*-factors based on *F*^2^ are statistically about twice as large as those based on *F*, and *R*- factors based on ALL data will be even larger.

### Fractional atomic coordinates and isotropic or equivalent isotropic displacement parameters (Å^2^)

**Table:**

	*x*	*y*	*z*	*U*_iso_*/*U*_eq_	
Pd1	0.01972 (3)	0.059770 (17)	0.31747 (2)	0.02267 (8)	
Cl1	−0.10981 (11)	−0.07263 (6)	0.31179 (8)	0.0316 (2)	
Cl2	−0.22196 (12)	0.13589 (7)	0.26969 (8)	0.0377 (2)	
N1	0.3194 (4)	0.0089 (2)	0.0758 (3)	0.0301 (7)	
N2	0.2618 (4)	0.1877 (2)	0.0729 (3)	0.0337 (7)	
N3	0.2348 (3)	−0.00482 (19)	0.3552 (2)	0.0230 (6)	
N4	0.1485 (4)	0.17256 (19)	0.3406 (2)	0.0276 (6)	
C1	0.3028 (4)	0.0517 (2)	0.1734 (3)	0.0232 (7)	
C2	0.2710 (4)	0.1409 (2)	0.1712 (3)	0.0266 (7)	
C3	0.2751 (5)	0.1441 (3)	−0.0234 (3)	0.0365 (9)	
H3	0.2666	0.1752	−0.0945	0.044*	
C4	0.3007 (5)	0.0556 (3)	−0.0224 (3)	0.0335 (8)	
H4	0.3052	0.0266	−0.0934	0.040*	
C5	0.3386 (4)	−0.0043 (2)	0.2797 (3)	0.0235 (7)	
C6	0.4750 (4)	−0.0569 (3)	0.2984 (3)	0.0312 (8)	
H6	0.5475	−0.0562	0.2450	0.037*	
C7	0.5064 (4)	−0.1105 (3)	0.3944 (3)	0.0333 (8)	
H7	0.5995	−0.1475	0.4075	0.040*	
C8	0.4000 (4)	−0.1094 (2)	0.4710 (3)	0.0290 (8)	
H8	0.4194	−0.1456	0.5380	0.035*	
C9	0.2662 (4)	−0.0558 (2)	0.4498 (3)	0.0257 (7)	
H9	0.1940	−0.0548	0.5033	0.031*	
C10	0.2571 (4)	0.1951 (2)	0.2742 (3)	0.0262 (7)	
C11	0.3524 (5)	0.2700 (2)	0.2987 (3)	0.0342 (9)	
H11	0.4274	0.2859	0.2507	0.041*	
C12	0.3370 (5)	0.3206 (3)	0.3926 (4)	0.0400 (10)	
H12	0.4024	0.3713	0.4109	0.048*	
C13	0.2252 (5)	0.2968 (3)	0.4605 (3)	0.0400 (10)	
H13	0.2128	0.3312	0.5257	0.048*	
C14	0.1324 (5)	0.2231 (2)	0.4324 (3)	0.0350 (9)	
H14	0.0550	0.2072	0.4785	0.042*	

### Atomic displacement parameters (Å^2^)

**Table:**

	*U*^11^	*U*^22^	*U*^33^	*U*^12^	*U*^13^	*U*^23^
Pd1	0.02314 (14)	0.02532 (15)	0.02103 (14)	0.00354 (10)	0.00798 (10)	0.00188 (10)
Cl1	0.0248 (4)	0.0329 (5)	0.0384 (5)	−0.0023 (4)	0.0089 (4)	0.0000 (4)
Cl2	0.0340 (5)	0.0461 (6)	0.0346 (5)	0.0158 (4)	0.0106 (4)	0.0100 (4)
N1	0.0340 (17)	0.0312 (17)	0.0285 (16)	−0.0009 (13)	0.0144 (13)	0.0009 (13)
N2	0.0414 (19)	0.0305 (17)	0.0296 (16)	−0.0011 (14)	0.0076 (14)	0.0063 (14)
N3	0.0219 (14)	0.0269 (16)	0.0204 (13)	0.0005 (11)	0.0044 (11)	0.0021 (12)
N4	0.0327 (17)	0.0243 (16)	0.0261 (15)	0.0050 (12)	0.0063 (13)	0.0035 (12)
C1	0.0202 (16)	0.0278 (18)	0.0220 (16)	−0.0005 (13)	0.0049 (13)	0.0035 (14)
C2	0.0271 (18)	0.0262 (19)	0.0273 (18)	−0.0015 (14)	0.0072 (15)	0.0023 (14)
C3	0.039 (2)	0.041 (2)	0.031 (2)	0.0001 (18)	0.0101 (17)	0.0107 (17)
C4	0.040 (2)	0.038 (2)	0.0253 (18)	−0.0008 (17)	0.0131 (16)	0.0034 (16)
C5	0.0219 (17)	0.0248 (18)	0.0244 (17)	−0.0024 (13)	0.0060 (14)	−0.0009 (14)
C6	0.0230 (18)	0.038 (2)	0.034 (2)	0.0046 (15)	0.0109 (15)	0.0073 (17)
C7	0.0258 (19)	0.036 (2)	0.035 (2)	0.0068 (16)	−0.0013 (15)	0.0091 (17)
C8	0.0270 (18)	0.032 (2)	0.0255 (18)	−0.0019 (15)	−0.0005 (14)	0.0081 (15)
C9	0.0274 (18)	0.0312 (19)	0.0178 (15)	0.0006 (14)	0.0023 (13)	0.0046 (14)
C10	0.0285 (18)	0.0199 (17)	0.0290 (18)	0.0014 (14)	0.0028 (15)	0.0035 (14)
C11	0.034 (2)	0.030 (2)	0.037 (2)	0.0003 (16)	0.0010 (16)	0.0043 (17)
C12	0.039 (2)	0.030 (2)	0.046 (2)	−0.0006 (17)	−0.0055 (19)	−0.0029 (18)
C13	0.055 (3)	0.027 (2)	0.032 (2)	0.0104 (18)	−0.0054 (19)	−0.0083 (17)
C14	0.051 (2)	0.027 (2)	0.0284 (19)	0.0096 (17)	0.0097 (17)	0.0009 (15)

### Geometric parameters (Å, °)

**Table:**

Pd1—N3	2.022 (3)	C4—H4	0.9500
Pd1—N4	2.026 (3)	C5—C6	1.377 (5)
Pd1—Cl1	2.2939 (10)	C6—C7	1.377 (5)
Pd1—Cl2	2.3037 (9)	C6—H6	0.9500
N1—C4	1.338 (4)	C7—C8	1.378 (5)
N1—C1	1.349 (4)	C7—H7	0.9500
N2—C3	1.335 (5)	C8—C9	1.370 (5)
N2—C2	1.347 (4)	C8—H8	0.9500
N3—C9	1.341 (4)	C9—H9	0.9500
N3—C5	1.351 (4)	C10—C11	1.394 (5)
N4—C10	1.347 (4)	C11—C12	1.372 (6)
N4—C14	1.353 (4)	C11—H11	0.9500
C1—C2	1.393 (5)	C12—C13	1.386 (6)
C1—C5	1.496 (5)	C12—H12	0.9500
C2—C10	1.488 (5)	C13—C14	1.374 (6)
C3—C4	1.374 (5)	C13—H13	0.9500
C3—H3	0.9500	C14—H14	0.9500
			
N3—Pd1—N4	87.89 (12)	C6—C5—C1	119.7 (3)
N3—Pd1—Cl1	88.08 (8)	C5—C6—C7	120.1 (3)
N4—Pd1—Cl1	173.45 (8)	C5—C6—H6	119.9
N3—Pd1—Cl2	178.07 (8)	C7—C6—H6	119.9
N4—Pd1—Cl2	90.98 (9)	C6—C7—C8	118.6 (3)
Cl1—Pd1—Cl2	93.19 (4)	C6—C7—H7	120.7
C4—N1—C1	117.1 (3)	C8—C7—H7	120.7
C3—N2—C2	117.2 (3)	C9—C8—C7	119.5 (3)
C9—N3—C5	119.7 (3)	C9—C8—H8	120.2
C9—N3—Pd1	119.4 (2)	C7—C8—H8	120.2
C5—N3—Pd1	120.5 (2)	N3—C9—C8	121.6 (3)
C10—N4—C14	119.5 (3)	N3—C9—H9	119.2
C10—N4—Pd1	122.7 (2)	C8—C9—H9	119.2
C14—N4—Pd1	117.7 (3)	N4—C10—C11	120.8 (3)
N1—C1—C2	120.8 (3)	N4—C10—C2	119.4 (3)
N1—C1—C5	113.0 (3)	C11—C10—C2	119.7 (3)
C2—C1—C5	125.8 (3)	C12—C11—C10	119.5 (4)
N2—C2—C1	121.2 (3)	C12—C11—H11	120.2
N2—C2—C10	113.4 (3)	C10—C11—H11	120.2
C1—C2—C10	125.3 (3)	C11—C12—C13	119.3 (4)
N2—C3—C4	121.6 (3)	C11—C12—H12	120.4
N2—C3—H3	119.2	C13—C12—H12	120.4
C4—C3—H3	119.2	C14—C13—C12	119.2 (4)
N1—C4—C3	121.9 (3)	C14—C13—H13	120.4
N1—C4—H4	119.1	C12—C13—H13	120.4
C3—C4—H4	119.1	N4—C14—C13	121.7 (4)
N3—C5—C6	120.4 (3)	N4—C14—H14	119.2
N3—C5—C1	119.9 (3)	C13—C14—H14	119.2
			
N4—Pd1—N3—C9	116.6 (3)	N1—C1—C5—C6	45.6 (4)
Cl1—Pd1—N3—C9	−58.2 (3)	C2—C1—C5—C6	−128.3 (4)
N4—Pd1—N3—C5	−70.4 (3)	N3—C5—C6—C7	0.0 (6)
Cl1—Pd1—N3—C5	114.7 (3)	C1—C5—C6—C7	−178.1 (3)
N3—Pd1—N4—C10	62.0 (3)	C5—C6—C7—C8	−0.7 (6)
Cl2—Pd1—N4—C10	−116.4 (3)	C6—C7—C8—C9	0.4 (6)
N3—Pd1—N4—C14	−112.7 (3)	C5—N3—C9—C8	−1.5 (5)
Cl2—Pd1—N4—C14	68.8 (3)	Pd1—N3—C9—C8	171.5 (3)
C4—N1—C1—C2	−1.3 (5)	C7—C8—C9—N3	0.8 (6)
C4—N1—C1—C5	−175.5 (3)	C14—N4—C10—C11	−0.3 (5)
C3—N2—C2—C1	3.7 (5)	Pd1—N4—C10—C11	−175.0 (3)
C3—N2—C2—C10	179.3 (3)	C14—N4—C10—C2	−178.2 (3)
N1—C1—C2—N2	−2.4 (5)	Pd1—N4—C10—C2	7.1 (4)
C5—C1—C2—N2	171.0 (3)	N2—C2—C10—N4	128.8 (3)
N1—C1—C2—C10	−177.4 (3)	C1—C2—C10—N4	−55.8 (5)
C5—C1—C2—C10	−4.0 (6)	N2—C2—C10—C11	−49.1 (5)
C2—N2—C3—C4	−1.4 (6)	C1—C2—C10—C11	126.2 (4)
C1—N1—C4—C3	3.7 (5)	N4—C10—C11—C12	1.0 (5)
N2—C3—C4—N1	−2.4 (6)	C2—C10—C11—C12	178.9 (3)
C9—N3—C5—C6	1.1 (5)	C10—C11—C12—C13	−0.9 (6)
Pd1—N3—C5—C6	−171.8 (3)	C11—C12—C13—C14	0.1 (6)
C9—N3—C5—C1	179.2 (3)	C10—N4—C14—C13	−0.5 (5)
Pd1—N3—C5—C1	6.3 (4)	Pd1—N4—C14—C13	174.4 (3)
N1—C1—C5—N3	−132.6 (3)	C12—C13—C14—N4	0.6 (6)
C2—C1—C5—N3	53.5 (5)		

### Hydrogen-bond geometry (Å, °)

**Table:**

*D*—H···*A*	*D*—H	H···*A*	*D*···*A*	*D*—H···*A*
C6—H6···Cl1^i^	0.95	2.83	3.445 (4)	124.
C11—H11···Cl1^ii^	0.95	2.82	3.629 (4)	143.

Symmetry codes: (i) *x*+1, *y*, *z*; (ii) −*x*+1/2, *y*+1/2, −*z*+1/2.
